# Porcine Recombinant NK-Lysin Inhibits the Growth and Metastasis of Murine Hepatocellular Carcinoma In Vivo

**DOI:** 10.3390/molecules30061234

**Published:** 2025-03-10

**Authors:** Kuohai Fan, Zhiwei Feng, Dahai Zhao, Xiaozhong Zheng, Wei Yin, Na Sun, Panpan Sun, Hongquan Li

**Affiliations:** 1 Shanxi Key Laboratory for Modernization of TCVM, College of Veterinary Medicine, Shanxi Agricultural University, Taigu 030801, China; fkhyxj@sxau.edu.cn (K.F.); fengzwi@163.com (Z.F.); 18434764821@163.com (D.Z.); xiaozhong.zheng@ed.ac.uk (X.Z.); dyxyyinwei@sxau.edu.cn (W.Y.); suna@sxau.edu.cn (N.S.); sunpcy0728@sxau.edu.cn (P.S.); 2 Laboratory Animal Center, Shanxi Agricultural University, Taigu 030801, China; 3 Medical Research Council (MRC) Centre for Inflammation Research, Queen’s Medical Research Institute, The University of Edinburgh, Edinburgh EH16 4TJ, UK

**Keywords:** prNK-lysin, anticancer activity, murine HCC, proliferation, metastasis

## Abstract

Porcine recombinant NK-lysin (prNK-lysin) has been shown to inhibit the proliferation and metastasis of hepatocellular carcinoma (HCC) cells in vitro. However, its effects on the proliferation and metastasis of HCC cells in vivo remain unclear. In this study, an allograft murine model using the murine HCC cell line Hepa1-6 was employed to investigate the anticancer effects of prNK-lysin. Initially, the in vitro anticancer efficacy of prNK-lysin was evaluated in Hepa1-6 cells, demonstrating that prNK-lysin effectively inhibited both proliferation and metastasis. These effects were mediated through the induction of oncosis and suppression of Fascin-1, MMP-2, and MMP-9 protein expressions. Subsequently, the in vivo anticancer efficacy of prNK-lysin was assessed using a mouse liver orthotopic implantation model and a lung metastasis model of Hepa1-6 cells in BALB/cA-nu mice. The administration of 13 mg/kg of prNK-lysin could inhibit tumor growth in the liver and metastasis to the lungs. Our results demonstrate that prNK-lysin possesses strong anti-HCC effects both in vitro and in vivo, with the induction of oncosis and the inhibition of Fascin-1, MMP-2, and MMP-9 protein expressions as potential molecular mechanisms for its anticancer activity.

## 1. Introduction

Hepatocellular carcinoma (HCC) is a major global health challenge due to its high morbidity and mortality rates. As of 2020, HCC accounted for approximately 830,180 deaths worldwide, making it the fourth leading cause of cancer-related deaths. The five-year survival rate for advanced HCC is around 18%, highlighting the aggressive nature of this malignancy [1]. Due to a significant resistance to chemotherapy, patients with advanced HCC have traditionally been treated with systemic therapy. The tyrosine kinase inhibitor Sorafenib has been the sole treatment option for advanced HCC since 2008 [2]. Therefore, the development of new drugs with specific cytotoxicity against HCC cells is clinically significant for HCC treatment.

Anticancer peptides (ACPs) are a class of naturally occurring peptides with anticancer activity [3]. Compared to traditional chemotherapy drugs, ACPs have advantages such as biocompatibility, efficient therapeutic efficacy, a low risk of drug resistance appearing in tumor cells, and limited or no toxicity against mammalian cells [4,5]. Additionally, ACPs have immunogenicity and low difficulty in synthesis and modification, with a short half-life in vivo, making them promising candidates for clinical anticancer drug development [4]. ACPs exert anticancer activity through membrane disruptive and non-membrane disruptive mechanisms including the mediation of the necrosis or apoptosis of cancer cells, the inhibition of angiogenesis, the recruitment of immune cells, and the activation of certain regulatory functional proteins [6]. In the Drug Bank Database, there are nearly 460 compounds targeting cancer, including 29 peptide- or polypeptide-based anticancer drugs. Most of the 29 peptides are still in the phases of preclinical or clinical trials. Currently, there are only five peptides approved for therapeutic purposes by regulatory agencies like the Food and Drug Administration (FDA) of the USA and European Medicines Agencies (EMAs) [7]. Therefore, there is an urgent need for the research and development of novel anticancer peptides.

NK-lysin is a type of granulysin originally isolated from swine intestinal tissues and identified as an effector peptide secreted by cytotoxic T lymphocytes (CTLs) and natural killer (NK) cells [8]. NK-lysin and its derivatives were demonstrated to have antimicrobial activity, anticancer activity, and immunomodulatory functions [9,10].

In our previous studies, the porcine recombinant NK-lysin (prNK-lysin) was expressed using a Pichia pastoris expression system [11], and demonstrated the ability to significantly inhibit the proliferation, migration, adhesion, and invasion of human HCC cells in vitro [12]. However, it is unclear whether the prNK-lysin can inhibit the growth and metastasis of HCC in vivo. This study aims to evaluate the efficacy of prNK-lysin in animal models of HCC and elucidate its underlying mechanisms of action.

## 2. Result

### 2.1. prNK-Lysin Inhibits the Proliferation of Hepa1-6 Cells In Vitro

**Figure 1:**
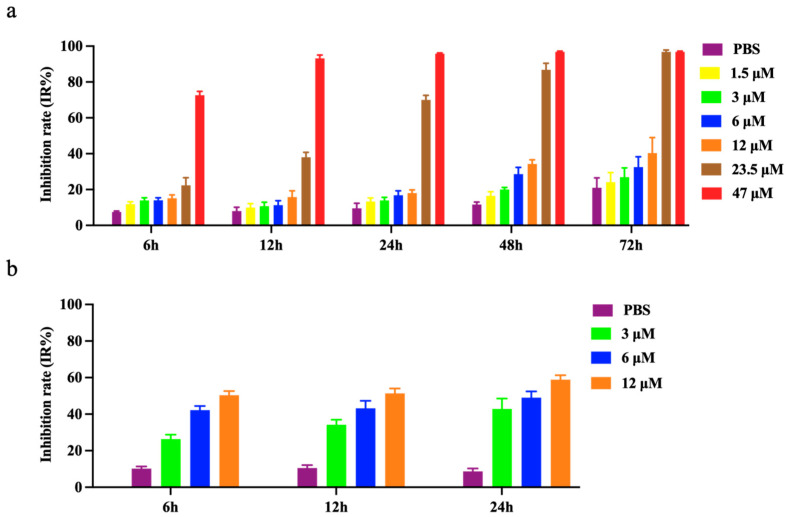
Inhibition rate of prNK-lysin on AML-12 hepatocytes and Hepa1-6 cells. (**a**) AML-12 hepatocytes were treated with the indicated concentration of prNK-lysin for 6 h, 12 h, 24 h, 48 h, and 72 h, (**b**) Hepa1-6 cells were treated with the indicated concentration of prNK-lysin for 6 h, 12 h, and 24 h.

The MTT assay results showed that the inhibitory rate of prNK-lysin at 12 μM on AML-12 hepatocytes proliferation did not exceed 20% within 24 h (Figure 1a), so 12 μM was the maximum safe concentration (MNTC) of prNK-lysin used for inhibiting AML-12 hepatocytes within a 24 h period. Subsequently, 3 μM, 6 μM, and 12 μM of prNK-lysin were used to treat Hepa1-6 cells within 24 h. The results demonstrate that compared to the control group, prNK-lysin significantly inhibited the proliferation of Hepa1-6 cells in a dose- and time-dependent manner (Figure 1b).

### 2.2. prNK-Lysin Induces Oncosis in Hepa1-6 Cells

**Figure 2:**
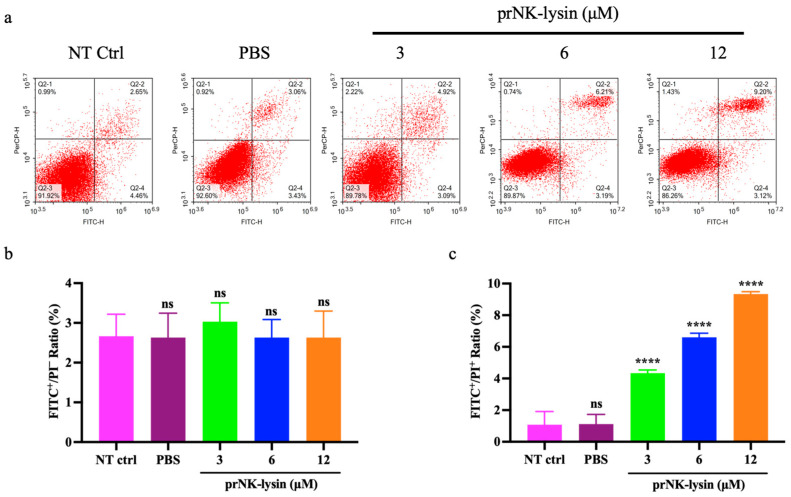
An Annexin V and PI double staining assay was used to analyze the effect of the treatment with prNK-lysin for 24 h. (**a**) Flow cytometry plots, (**b**) the bar statistics of the FITC^+^/PI^−^ cells rate, (**c**) the bar statistics of the FITC^+^/PI^+^ cells rate. The ns means not statistically significant, and **** indicates *p* < 0.0001.

**Figure 3:**
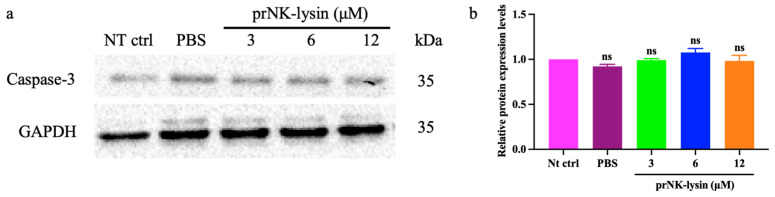
The expression of Caspase-3 in the Hepa1-6 cells treated with different concentrations of prNK-lysin for 24 h. (**a**) Images of Western blotting, (**b**) the bar statistics of expression of Caspase-3. Caspase-3 band intensities were quantified by Image J (v1.8.0) after GAPDH normalization. The ns means not statistically significant.

Double staining with Annexin V-FITC/PI was used to evaluate the cell apoptosis induced by prNK-lysin in Hepa1-6 cells. The results showed that prNK-lysin significantly increased the number of FITC^+^/PI^+^ cells, but not FITC^+^/PI^-^ cells (Figure 2). Additionally, the expression of Caspase-3 in the group treated with prNK-lysin did not exhibit a significant alteration in comparison with the control group (Figure 3). These results indicated that prNK-lysin induced non-apoptotic cell death.

**Figure 4:**
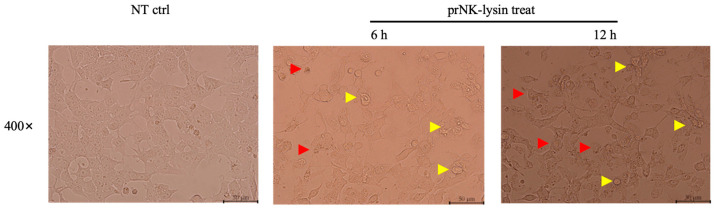
The morphological changes in the Hepa1-6 cells treated with 12 μM of prNK-lysin for 6 h and 12 h were observed by Inverted Microscope (Scale bar = 10 μm. Yellow arrow →: membrane surface blebs, Red arrow →: cellular swelling).

**Figure 5:**
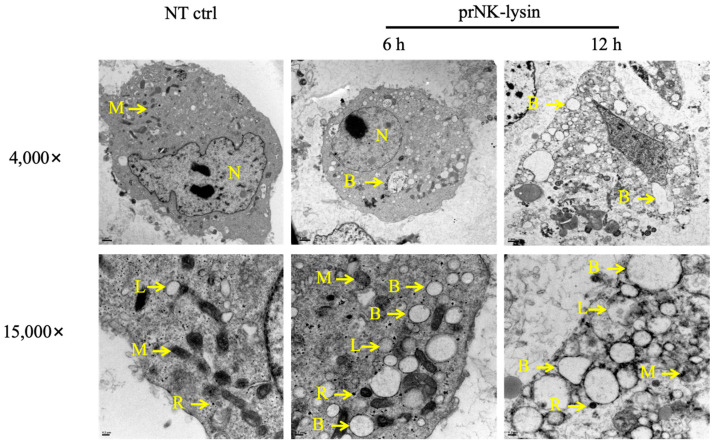
The morphological changes in the Hepa1-6 cells treated with 12 μM of prNK-lysin for 6 h and 12 h were observed by Transmission Electron Microscope (Scale bar = 1 μm and 0.2 μm. N: Nuclear, M: Mitochondria, R: Ribosome, L: Lysosome, B: Blebbing).

**Figure 6:**
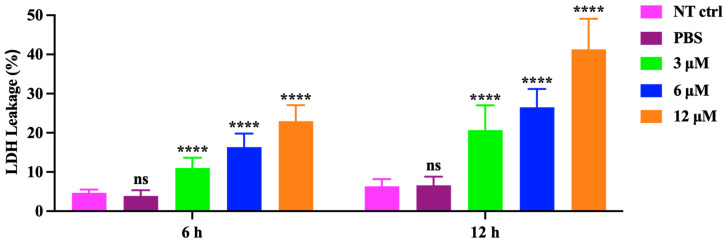
Lactate dehydrogenase (LDH) release in Hepa1-6 cells treated with different concentrations of prNK-lysin for 6 h and 12 h. The ns means not statistically significant, and **** indicates *p* < 0.0001.

**Figure 7:**
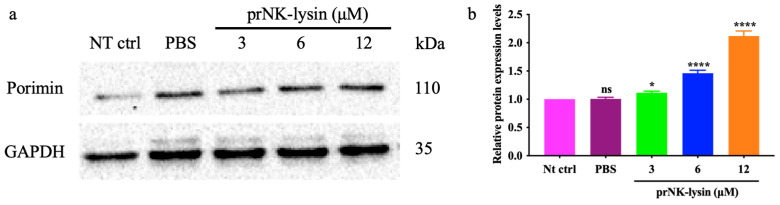
The expression of Porimin in the Hepa1-6 cells treated with different concentrations of prNK-lysin for 24 h. (**a**) Images of Western blotting, (**b**) the bar statistics of expression of Porimin. Porimin band intensities were quantified by Image J (v1.8.0) after GAPDH normalization. The ns means not statistically significant, * indicates *p* < 0.05, and **** indicates *p* < 0.0001.

We also investigated the changes of cell morphology, the levels of extracellular lactate dehydrogenase (LDH), and porimin expression. Compared to the control group, cellular swelling and membrane surface blebs were observed in the prNK-lysin treatment groups (Figure 4). Numerous vacuoles, mitochondrial swelling, and karyolysis were detected inside the cell treated with prNK-lysin (Figure 5). Compared to the control group, prNK-lysin treatment significantly increased the level of LDH released (Figure 6) and porimin expression (Figure 7) by Hepa1-6 cells.

### 2.3. prNK-Lysin Inhibits Metastasis of Hepa1-6 Cells In Vitro

**Figure 8:**
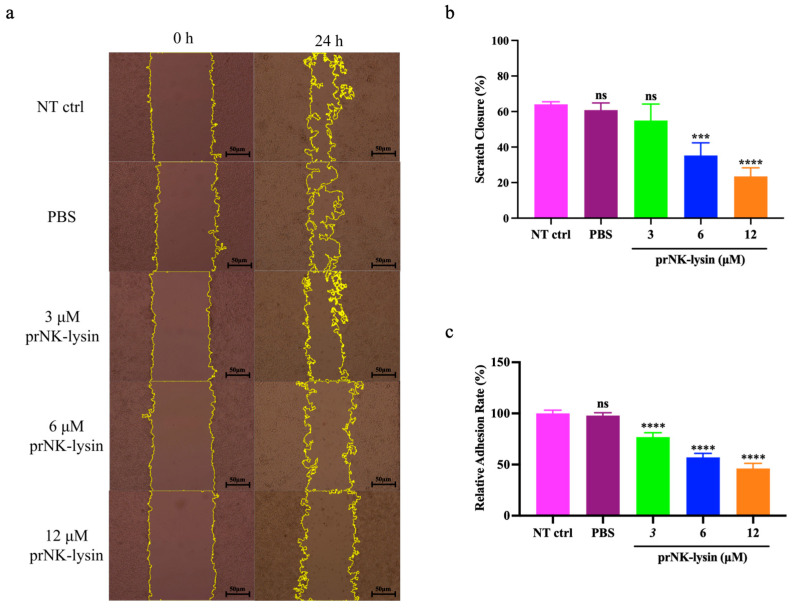
The scratch healing and adhesion of the Hepa1-6 cells treated with different concentrations of prNK-lysin for 24 h was observed and analyzed. (**a**) Images of scratch healing of the Hepa1-6 cells (scale = 50 μm), (**b**) the bar statistics of scratch healing of the Hepa1-6 cells, (**c**) the bar statistics of adhesion of the Hepa1-6 cells. The ns means not statistically significant, *** indicates *p* < 0.001, and **** indicates *p* < 0.0001.

**Figure 9:**
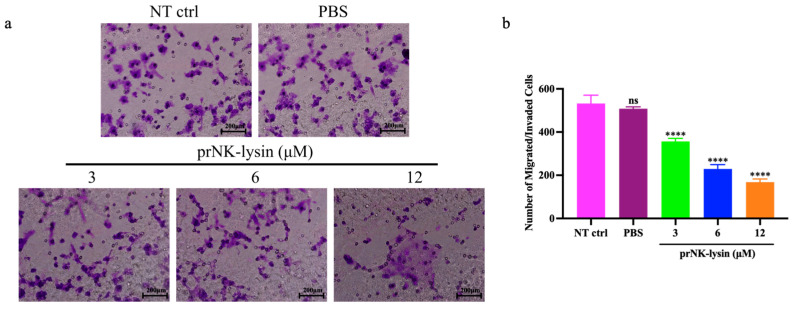
The migration and invasion of the Hepa1-6 cells treated with different concentrations of prNK-lysin for 24 h was observed and analyzed. (**a**) Images of migration and invasion of the Hepa1-6 cells (scale = 200 μm), (**b**) the bar statistics of migration and invasion of the Hepa1-6 cells. The ns means not statistically significant, and **** indicates *p* < 0.0001.

The results showed that prNK-lysin treatment for 24 h significantly decreased the migration (Figure 8a,b), adhesion (Figure 8c), and invasion ability (Figure 9) of the Hepa1-6 cells. These results demonstrated that prNK-lysin treatment significantly inhibits the metastasis of Hepa1-6 cells in vitro in a dose-dependent manner.

**Figure 10:**
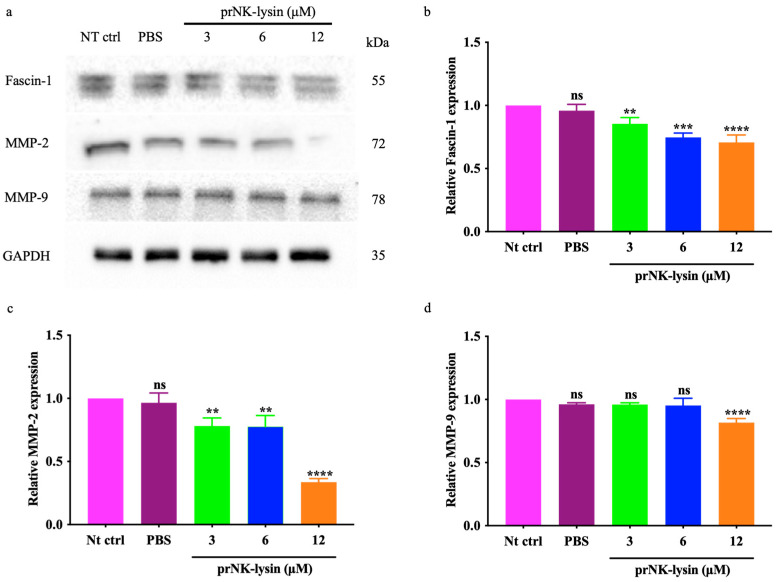
The expressions of Fascin-1, MMP-2, and MMP-3 in the Hepa1-6 cells treated with different concentrations of prNK-lysin for 24 h. (**a**) Images of Western blotting, (**b**–**d**) the bar statistics of expression of Fascin-1, MMP-2 and MMP-9. Fascin-1, MMP-2, and MMP-9 band intensities were quantified by Image J (v1.8.0) after GAPDH normalization. The ns means not statistically significant, ** indicates *p* < 0.01, *** indicates *p* < 0.001, and **** indicates *p* < 0.0001.

Elevated levels of Fascin-1 are linked to increased tumor invasiveness, affecting metastasis severity and leading to poor prognoses and shorter overall survival times. Matrix metalloproteinases (MMPs, such as MMP-1, MMP-2, and MMP-9) facilitate the breakdown of proteins in the extracellular matrix, enabling cell migration [13]. The level of Fascin-1, MMP-2, and MMP-3 in the Hepa1-6 cells were detected using Western blotting. The results showed that the expressions of Fascin-1, MMP-2, and MMP-3 were down-regulated in the Hepa1-6 cells treated with prNK-lysin (Figure 10). This indicated that prNK-lysin inhibits the metastasis of Hepa1-6 cells by inhibiting Fascin-1, MMP-2, and MMP-3 expressions.

### 2.4. prNK-Lysin Inhibits the Growth of Hepa1-6 Cells in Mouse Liver Orthotopic Implantation Model

**Figure 11:**
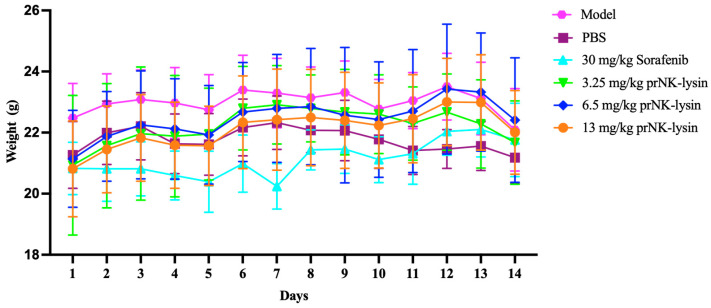
Changes in body weight observed using mouse liver orthotopic implantation model.

**Figure 12:**
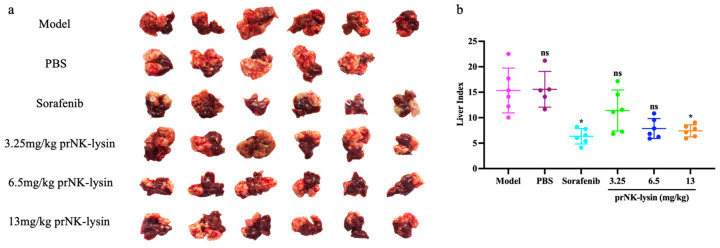
The growth of Hepa1-6 cells in mouse liver orthotopic implantation model. (**a**) Images of tumor and liver tissues observed under mouse liver orthotopic implantation model, (**b**) The bar statistics of liver index. The ns means not statistically significant, and * indicates *p* < 0.05.

**Figure 13:**
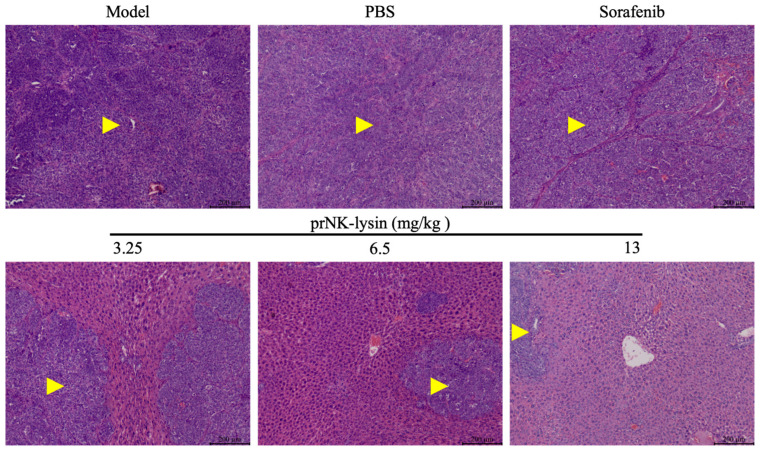
Histology of tumor and liver tissue staining with HE (Scale bar = 200 μm, Yellow arrow →: Cancerous tissue).

The effect of prNK-lysin on HCC growth in vivo was evaluated using the mouse liver orthotopic implantation model. As shown in Figure 11, there was no significant difference in body weight among all of the groups. The liver indices of the Sorafenib group and the 13mg/kg prNK-lysin group were significantly lower than the other groups (Figure 12). The liver tissues in HE staining showed that in the model group and the PBS group, the liver lobule structure was unclear, the hepatic cords were disorganized, the hepatic sinusoids were reduced or even disappeared, some hepatocytes were necrotic, the sizes of hepatocyte nuclei varied, and the liver cancer cells were distributed in diffuse form, most of them appearing as immature cells. In the 3.25 mg/kg prNK-lysin group, the hepatic sinusoids disappeared, and the liver cancer cells were diffusely distributed. In the 6.5 mg/kg prNK-lysin group, the hepatic sinusoids were smaller, with a clear boundary between the normal hepatocytes and the liver cancer cells. In the 13 mg/kg prNK-lysin group and the Sorafenib group, the liver lobule structure was clear, the hepatic cords were orderly arranged, the hepatic sinusoids were normal, the sizes of hepatocyte nuclei were uniform, and the distribution of the liver cancer cells was reduced (Figure 13). The results above indicated prNK-lysin inhibited the growth of Hepa1-6 cells in vivo.

### 2.5. prNK-Lysin Inhibits the Metastasis of Hepa1-6 Cells in Mouse Lung Metastasis Model

**Figure 14:**
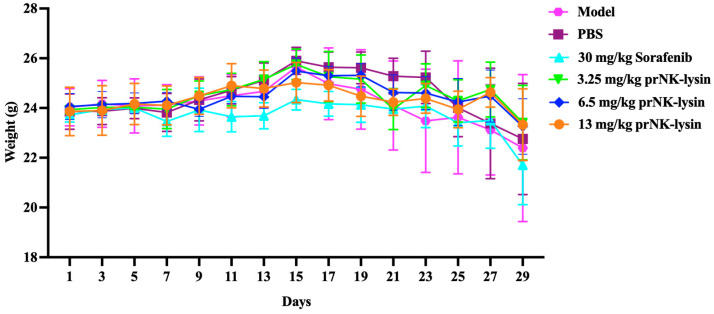
Changes in the body weight under the mouse lung metastasis model.

**Figure 15:**
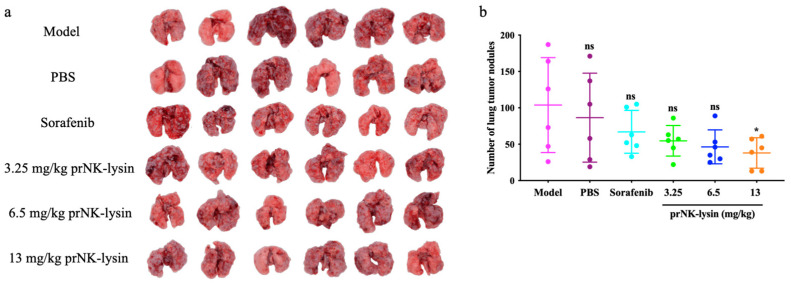
The metastasis of Hepa1-6 cells in mouse lung metastasis model. (**a**) Images of lung tissues under the mouse lung metastasis model, (**b**) The bar statistics of number of lung tumor nodules. The ns means not statistically significant, and * indicates *p* < 0.05.

**Figure 16:**
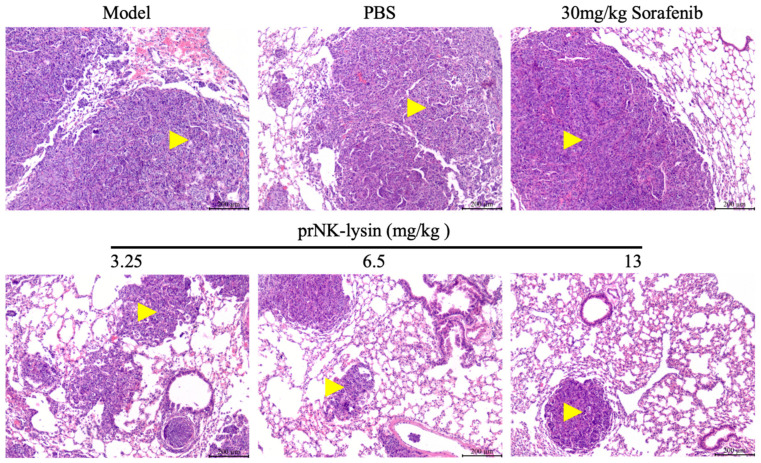
Histology of tumor and lung tissue staining with HE (Scale bar = 200 μm, Yellow arrow →: Cancerous tissue).

The prNK-lysin effect on HCC metastasis in vivo was studied in a lung metastasis model of a mouse injected with Hepa1-6 cells. As shown in Figure 14, there was no significant difference in body weight between all of the groups. The number of tumor nodules in the PBS group, the Sorafenib group, as well as the 3.25 mg/kg, 6.5 mg/kg, and 13 mg/kg prNK-lysin groups were less than the model group, but there were no statistically significant differences between the groups except for the 13 mg/kg prNK-lysin group (Figure 15). The HE staining of liver tissues showed that the cancer nests in the model group, PBS group, and Sorafenib group occupied almost all of the lung tissue and the boundary between the tumor and normal lung tissue was blurry. In the 3.25 mg/kg and 6.5 mg/kg prNK-lysin groups, the cancer nest area was smaller than that of the model group, but it had blurred boundary with the normal lung tissue. The cancer nest area of the 13 mg/kg prNK-lysin group was obviously smaller than the other groups and had a clear boundary with the normal lung tissue (Figure 16). This indicated that prNK-lysin inhibited the metastasis of Hepa1-6 cells in vivo.

## 3. Discussion

In the previous studies, we found that prNK-lysin could inhibit the proliferation and metastasis of human HCC cells when a non-toxic concentration of prNK-lysin to normal hepatocytes L-02 in vitro was applied [12]. In this study, we demonstrated the inhibitory effect of prNK-lysin on murine HCC cells, Hepa1-6, with a non-toxic concentration of AML-12 hepatocytes in vitro and in vivo.

Apoptosis has traditionally been a key target for anticancer therapy. The recombinant NK-lysin could induce apoptosis of Jurkat cells [14]. However, our results indicated that the cell death of Hepa1-6 cells induced by the prNK-lysin was non-apoptotic cell death. The Hepa1-6 cells treated with prNK-lysin exhibited distinctive features, including cellular swelling, membrane surface blebs with vacuoles, mitochondrial swelling, and karyolysis inside the cells. The morphological manifestations of the Hepa1-6 cells were not typical of apoptosis. Oncosis is a form of death characterized by cell swelling and karyolysis [15]. Its morphological characteristics include cell enlargement, swelling, cell vesicles, a lack of organelles in the vesicles, the destruction of cell membrane integrity, the swelling of the endoplasmic reticulum, the swelling of mitochondria, a swollen nuclear membrane, dispersion, and the agglutination of chromatin [16,17,18]. Additionally, LDH leakage, an indicator of the integrity of the cell membrane, is associated with oncosis [19], and the porimin is specifically expressed on the surface of oncotic cells [20]. Furthermore, prNK-lysin was found to induce LDH leakage and elevate porimin levels in Hepa1-6 cells, indicating that prNK-lysin-induced cell death is oncosis.

Metastasis involves the spread of cancer cells from the primary tumor to the surrounding tissues and other distant organs and is the primary cause of cancer morbidity and mortality. Inhibiting cancer metastasis plays a crucial role in enhancing the effectiveness of cancer treatment, improving patient outcomes, and maintaining a better quality of life. Fascin-1 is a protein closely related to the cross-linking of filaments in actin-rich protrusions [21]. Its overexpression is observed in metastatic cancer. Hence, Fascin-1 is believed to promote the migration and invasion of cancer cells [22,23], and has been considered a clinical prognostic marker of metastatic tumors [24]. Gelatin is one of the major components of the extracellular matrix (ECM) around a cancer cell. Since ECM acts as a biochemical and biophysical barrier for cancer cell migration and invasion into the blood/lymphatic vessels, ECM degradation must be preceded for cancer metastasis. There are more than 20 matrix metalloproteinases (MMPs), which are major enzymes that degrade the components of an ECM. Among them, MMP-2 and MMP-9 are two major gelatin-degrading enzymes, or gelatinases [25]. In the present study, we performed selective assays which were related to cancer cell migration and invasion in murine HCC cells to clarify the anti-metastatic effects of prNK-lysin. The prNK-lysin significantly inhibited the migration, adhesion, and invasion of the Hepa1-6 cells. The expressions of Fascin-1, MMP-2, and MMP-9 were also suppressed by the prNK-lysin treatment. Subsequent animal experiments provided further validation of prNK-lysin’s inhibitory effect on murine HCC growth and metastasis. The anticancer effect of prNK-lysin was better than that seen in the Sorafenib group.

ACPs exert their anticancer effects by destroying the structure of cell membranes, inducing necrosis or apoptosis, and inhibiting angiogenesis and immune regulation [6]. In our previous study, we demonstrated that prNK-lysin exerts its anticancer effect on human HCC cells in vitro through membrane disruption [11] and the inhibition of Fascin-1 expression, which regulates the Wnt/β-catenin signaling pathway by inducing β-catenin degradation and subsequently results in the suppression of MMP-2 and MMP-9 expression [12]. In this study, inducing oncosis has been proven to be one of the anticancer mechanisms of prNK-lysin, except in relation to the suppression of Fascin-1, MMP-2, and MMP-9 expression. It reports that NK-lysin can act as an immunomodulator, inducing the expression of immune genes and modulating the immune responses [9]. However, whether prNK-lysin has an immunomodulatory function needs further investigation. Like other peptides, prNK-lysin is susceptible to proteolytic degradation in vivo. Strategies such as peptide cyclization or encapsulation in nanoparticles may improve its stability. The potential for immune responses against prNK-lysin requires further investigation.

## 4. Material and Methods

### 4.1. prNK-Lysin, Cells and Cell Culture

The prNK-lysin was prepared in our laboratory according to our previously established method [11].

The Alpha mouse liver 12 (AML-12) hepatocyte was purchased from Servicebio Technology (Wuhan, Hubei, China). Murine hepatocellular carcinoma cell Hepa1-6 was purchased from the Institute of Basic Medical Sciences of the Chinese Academy of Medical Sciences. These cells were maintained in Dulbecco’s Minimum Essential Medium (DMEM) and were supplemented with 10% Fetal Bovine Serum (FBS), 100 U/mL of penicillin, and 100 μg/mL of streptomycin, at 37 °C in a 5% CO_2_ incubator.

### 4.2. Cell Viability Assay

AML-12 hepatocytes and Hepa1-6 cells were seeded into a 96-well cell culture plate with 5 × 10^3^ cells/well for overnight growth and then treated with prNK-lysin at different concentrations at different time periods. Then, the culture supernatant was discarded, and the cells were incubated with 30 μL of MTT reagent for 4 h, and further incubated for 30 min by adding 100 μL of DMSO reagent. Subsequently, the absorbance value was measured at 490 nm using a SpectraMax i3x Multi-Mode Microplate Reader (Molecular Devices, San Jose, CA, USA). The inhibition rate of cell proliferation (IR) was calculated as follows: IR = [(NT ctrl OD value − experimental group OD value)/NT ctrl OD value] × 100%.

### 4.3. Cell Death Detection

The Hepa1-6 cells were seeded into a 6-well cell culture plate with 5 × 10^4^ cells/well for overnight growth, and then treated with prNK-lysin at different concentrations for 24 h. The cells were collected using trypsin digestion without EDTA, washed with cold PBS, and stained in the dark following the instructions provided in Annexin V-FITC/PI Apoptosis Detection Kit. Cell death was detected using FACScan Flow Cytometer (Becton Dickinson, Franklin Lakes, NJ, USA).

### 4.4. Light Microscope

The Hepa1-6 cells were seeded into a 96-well cell culture plate with 5 × 10^3^ cells/well for overnight growth and then treated with 12 μM of prNK-lysin for 6 and 12 h. The cells were monitored, and images were captured using an inverted microscope (Olympus, Shinjuku, Tokyo, Japan).

### 4.5. Transmission Electron Microscopy

The Hepa1-6 cells were seeded into a 100 mm cell culture plate with 6 × 10^5^ cells/well for overnight growth, and then treated with 12 μM of prNK-lysin for 6 and 12 h. The cells were washed with PBS and fixed with an electron microscope fixative. The cell samples were placed on copper mesh for staining, with observations performed using H-7650 Transmission Electron Microscopy (Hitachi, Chiyoda, Tokyo, Japan).

### 4.6. LDH Detection

The Hepa1-6 cells were seeded into a 96-well cell culture plate with 5 × 10^3^ cells/well for overnight growth and then treated with prNK-lysin at different concentrations for 6 and 12 h. The supernatant was collected following the instructions provided in the Lactate Dehydrogenase (LDH) Assay Kit, and the LDH level was evaluated by measuring the absorbance at 490 nm using a SpectraMax i3x Multi-Mode Microplate Reader (Molecular Devices, San Jose, CA, USA).

### 4.7. Scratch Assay

The Hepa1-6 cells were seeded into a 6-well cell culture plate with 5 × 10^4^ cells/well and incubated until they reached 100% confluence. A scratch was created manually through the cell monolayer using a 100 μL pipette tip. Cells and debris were rinsed with PBS. Then, the cells were treated with prNK-lysin at different concentrations. Images of the scratch areas were taken immediately after making a scratch (0 h) and at 24 h for further incubation. The width of a scratch was determined based on the images taken and the rate of cell migration was calculated using Image J (v1.8.0) (NIH Image, Bethesda, MD, USA). The percentage of scratch closure was computed using the formula: Scratch Closure (%) = [(Initial Scratch Area − Final Scratch Area)/Initial Scratch Area] × 100%.

### 4.8. Adhesion Assay

The Hepa1-6 cells were seeded into a 6-well cell culture plate with 5 × 10^4^ cells/well overnight and then treated with prNK-lysin at different concentrations for 24 h. A total of 100 µL of cell suspension treated with prNK-lysin was added into the 96-well cell culture plate coated with Matrigel and incubated for 1 h. The wells were washed with PBS to remove the non-adherent cells. The adherent cells were incubated with 30 μL of MTT reagent for 4 h, and further incubated for 30 min by adding 100 μL of DMSO reagent. Subsequently, the absorbance value was measured at 490 nm using a SpectraMax i3x Multi-Mode Microplate Reader (Molecular Devices, San Jose, CA, USA). The percentage of relative adhesion was computed using the formula: Relative Adhesion Rate (%) = OD of Experimental Group/OD of Control Group × 100%.

### 4.9. Migration and Invasion Assay

The upper chamber of the Transwell inserts was coated with a thin layer of Matrigel and incubated at 37 °C for 2 h to allow the Matrigel to solidify. A total of 200 μL of an FBS-free DMEM containing 4 × 10^4^ cells and prNK-lysin at different concentrations was added into the upper chamber of the Matrigel-coated Transwell insert. A total of 500 µL of a DMEM containing 20% FBS was added to the lower chamber. The Transwell inserts were in the lower chamber and incubated for 24 h. The non-invaded cells were removed from the upper surface of the insert using a cotton swab. The cells that invaded through the Matrigel were fixed to the lower surface of the insert with 4% paraformaldehyde for 10 min. The cells were stained with crystal violet for 30 min, then rinsed with PBS. Counted The stained cells were counted using an inverted microscope (Olympus, Shinjuku, Tokyo, Japan).

### 4.10. Western Blot Analysis

The Hepa1-6 cells were seeded into a 100 mm cell culture plate with 6 × 10^5^ cells/well for overnight growth and then treated with prNK-lysin of different concentrations for 24 h. The cells were lysed using an RIPA lysis buffer containing 1% cocktail and 1% PMSF. The protein concentration was determined using a BCA Protein Kit. The protein samples were separated by SDS-PAGE and transferred to the 0.45 μm PVDF membrane. Then, the membrane was blocked with 10% skimmed milk for 2 h. The membranes were incubated with primary antibodies overnight at 4 °C, including a Caspase-3 Antibody (1:1000; CST, Danvers, MA, USA), a Porimin Antibody (1:500; NOVUS, Centennial, CO, USA), a Fascin-1 Antibody (1:25,000; ABCAM, Waltham, MA, USA), an MMP-2 Antibody (1:2500; ABCAM, Waltham, MA, USA), an MMP-9 Antibody (1:5000; ABCAM, Waltham, MA, USA), and a GAPDH Antibody (1:30,000; Proteintech, Wuhan, China), and then the membranes were incubated with secondary antibodies including Goat anti-rabbit IgG-HRP (1:20,000, Cwbio, Beijing, China) and Goat anti-mouse IgG-HRP (1:20,000, Absin, Shanghai, China) at room temperature by shaking for 1 h. Subsequently, the target protein was visualized using an enhanced chemiluminescence reagent (MeilunBio, Dalian, China). The target protein band intensities were quantified by Image J (v1.8.0) (NIH Image, Bethesda, MD, USA) after GAPDH normalization.

### 4.11. Animals

The animal experiments were approved by the Institutional Animal Care and Use Committee of Shanxi Agricultural University (approval document No. 2022NM.ZA-005010001 for the mouse liver orthotopic implantation model and SXAU-EAW-2022M.FC.008011002 for the mouse lung metastasis model). All animals received care according to the standards outlined in the National Standard Animal Experiment Endpoint Evaluation Guidelines of the People’s Republic of China. Male BALB/cA-nu mice were purchased from Beijing HFK Bioscience Co., Ltd. (animal license #: SCXK Beijing 2024-0003, China). All animals were fed under standard SPF-grade conditions at 25 °C with 50% relative humidity, and with free access to drinking water.

### 4.12. Establishment of Mouse Orthotopic Model by Seeding Hepa1-6 Cells into Mouse Liver

The 7-week-old male mice were anesthetized using isoflurane. The mice were placed in a supine position with the abdominal area sterilized, then a subcostal incision was made to expose the liver. The Hepa1-6 cells (5 × 10^5^ cells/mouse) were injected into the left lobe of the liver using a fine-gauge needle. The abdominal muscle layer was closed with absorbable sutures and the skin was closed with non-absorbable sutures. The mice were closely monitored until they fully recovered from the anesthesia. Following 24 h post-surgery, the mice were randomly divided into the model group, PBS group, 30 mg/kg Sorafenib group, 3.25 mg/kg prNK-lysin group, 6.5 mg/kg prNK-lysin group, and 13 mg/kg prNK-lysin group (*n* = 6). The Sorafenib was dissolved in 5% DMSO and 95% PBS, and the prNK-lysin was dissolved in PBS. Except for the model group, PBS or drugs were administered into the mice of the other groups by intraperitoneal injection once a day for 14 consecutive days. The mice were monitored daily for weight loss, health, and welfare. All the mice were humanely euthanized by CO_2_ inhalation on the 15th day, and their livers were harvested and weighed. The liver index was calculated using the following formula: Liver Index = [Liver Weight (g)/Body Weight (g)] × 100%.

### 4.13. Establishment of Mouse Lung Metastasis Model

The 5-week-old male mice were anesthetized using isoflurane for cell injection. The Hepa1-6 cells (2 × 10^5^ cells/mouse) were injected into the tail vein using a fine-gauge needle for 5 consecutive days. On the 6th day, the mice were randomly divided into the model group, PBS group, 30 mg/kg Sorafenib group, 3.25 mg/kg prNK-lysin group, 6.5 mg/kg prNK-lysin group, and 13 mg/kg prNK-lysin group (*n* = 6). Except for the model group, PBS or drugs were administered into the mice of the other groups by intraperitoneal injection once a day for 14 consecutive days. The mice were monitored daily for weight loss, health, and welfare. All the mice were humanely euthanized by CO_2_ inhalation on the 15th day, and their lungs were harvested and weighed. The number of lung tumor nodules was counted by manual counting.

### 4.14. Histopathology Analysis

The livers and lungs of the mice were collected, fixed, and processed onto paraffin-embedded (FFPE) blocks. The hematoxylin–eosin (HE) staining on the mice livers and lungs was performed. The stained slides were photographed and analyzed using an upright microscope (Leica, Wetzlar, Hesse, Germany).

### 4.15. Statistical Analysis

Data were expressed as the mean ± standard deviation (SD). Statistical analyses were performed with one-way ANOVA comparing the samples with their respective control using GraphPad Prism 7.0 software (GraphPad Software Inc., San Diego, CA, USA). A significant difference was considered when *p* < 0.05 (* *p* < 0.05, ** *p* < 0.01, *** *p* < 0.001, **** *p* < 0.0001).

## 5. Conclusions

This study demonstrates that prNK-lysin has anti-HCC activity in vivo and prNK-lysin is a potential candidate as a therapeutic option for HCC, offering a novel avenue for addressing this challenging malignancy.

## Data Availability

The data will be available upon request.
